# TPI1 Loss Triggers a Metabolite‐Driven Mitochondrial Redox Vulnerability via the SARM1–cADPR–Ca^2+^ Axis

**DOI:** 10.1002/advs.76614

**Published:** 2026-07-17

**Authors:** Chunyu Liu, Shun Wu, Chuang Wang, Zirui Zhou, Tianwei Cai, Wen Tao, Shidong Zuo, Chi Zhang, Yuhao Dong, Yi Feng, Qingbo Huang, Baojun Wang, Xin Ma, Haoli Ma, Xu Zhang, Yan Huang

**Affiliations:** ^1^ Senior Department of Urology Chinese PLA General Hospital Beijing China; ^2^ Medical Schhool of PLA Beijing China; ^3^ Department of Biological Repositories Zhongnan Hospital of Wuhan University Wuhan China; ^4^ Department of Urology Renmin Hospital of Wuhan University Wuhan China

**Keywords:** cADPR, calcium, cancer, senescence, TPI1

## Abstract

Cellular senescence is a stable cell cycle arrest program with important therapeutic implications in cancer, yet how metabolic perturbations are translated into redox‐dependent senescence remains incompletely understood. Here, we identify triosephosphate isomerase 1 (TPI1) as a critical regulator of senescence in clear cell renal cell carcinoma (ccRCC) through a customized CRISPR‐Cas9 metabolic screen. TPI1 depletion induces a robust senescence phenotype characterized by mitochondrial redox imbalance, DNA damage, and stable growth arrest. Mechanistically, loss of TPI1 leads to accumulation of dihydroxyacetone phosphate (DHAP), which engages a SARM1‐dependent signaling pathway, resulting in increased cyclic ADP‐ribose (cADPR) production and intracellular Ca^2+^ release. This cADPR‐Ca^2+^ axis drives mitochondrial ROS (mtROS) generation, thereby promoting DNA damage and activation of the p53‐p21 pathway to enforce senescence. Pharmacological or genetic attenuation of calcium signaling, or mitochondrial ROS partially rescues these phenotypes, indicating that calcium‐dependent redox stress is required for senescence induction. Importantly, this metabolic–redox signaling cascade is conserved across multiple cancer types. Collectively, our findings define a previously unrecognized TPI1–SARM1–cADPR–Ca^2+^ axis that links glycolytic metabolite accumulation to mitochondrial redox stress and cellular senescence, highlighting a metabolite‐driven redox vulnerability that may be therapeutically exploitable in cancer.

## Graphical abstract

Metabolite‐driven redox stress governs cancer cell senescence. TPI1 deficiency elevates DHAP, initiating SARM1‐dependent cADPR‐Ca^2+^ release. Mitochondrial ROS surge subsequently induces DNA damage and senescence, offering a conserved therapeutic target in multiple cancers.

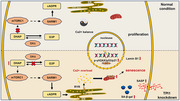

## 1 Introduction

Cellular senescence is a state of stable cell cycle arrest triggered by diverse endogenous and exogenous stresses. It manifests as a heterogeneous set of phenotypes, including persistent reactive oxygen species (ROS) accumulation, DNA damage responses (DDR), and the senescence‐associated secretory phenotype (SASP). In malignant cells, the induction of senescence represents a promising anticancer strategy, enabling durable proliferative arrest and promoting immune‐mediated clearance [1]. Therapeutically, senescence can be triggered by chemotherapy, radiotherapy, or targeted agents; however, efficacy is often constrained by heterogeneous responses and the potentially deleterious effects of SASP [2, 3]. As a predominant intracellular source of ROS [4], mitochondria‐derived ROS (mtROS) function at the intersection of metabolism and signaling, where they not only arise as byproducts of oxidative metabolism but also actively drive DNA damage‐particularly double‐strand breaks‐thereby triggering DDR activation and enforcing cell cycle arrest to consolidate the senescent phenotype [5, 6, 7]. Despite increasing recognition of the intimate link between cellular metabolism and senescence, the specific metabolic nodes that initiate and execute senescence programs in cancer cells remain largely undefined, especially in the context of metabolite‐driven signaling cascades [8, 9, 10].

Triosephosphate isomerase 1 (TPI1), a key glycolytic enzyme, catalyzes the reversible interconversion between glyceraldehyde‐3‐phosphate (GAP) and dihydroxyacetone phosphate (DHAP), and under physiological conditions preferentially drives DHAP toward GAP [11]. TPI1 deficiency is an autosomal recessive multisystem disorder characterized by reduced enzymatic activity and consequent DHAP accumulation [12]. The E104D mutation is the most prevalent pathogenic variant, with clinical manifestations including hemolytic anemia and neurological dysfunction, frequently leading to premature death [13]. A TPI1‐deficient Drosophila model recapitulates key disease phenotypes and has been extensively used to investigate disease mechanisms. Notably, neither this model nor TPI1‐deficient patients exhibit overt defects in ATP production, indicating that classical bioenergetic failure is unlikely to be the primary driver of pathology [14, 15]. Instead, emerging evidence suggests that TPI1 deficiency is associated with redox imbalance, as indicated by increased levels of oxidized glutathione and a more oxidized mitochondrial state [14]. In parallel, TPI1 is frequently overexpressed in multiple cancers and is associated with poor clinical outcomes [11, 13, 16, 17]. Moreover, DHAP has been reported to activate mTORC1 signaling, induce cell cycle arrest, and promote ROS production [18, 19, 20]. These observations collectively suggest that perturbations in TPI1 activity may rewire metabolic and redox signaling. However, whether and how such metabolic alterations are coupled to senescence programs in cancer cells remains largely unknown.

Cyclic ADP‐ribose (cADPR) is a key second messenger generated by ADP‐ribosyl cyclases, including Cluster of Differentiation 38 (CD38) and Sterile Alpha And TIR Motif Containing 1 (SARM1), and plays a central role in mobilizing intracellular Ca^2+^ stores through activation of ryanodine receptors on the endoplasmic reticulum (ER) [21, 22]. Through its regulation of calcium release, cADPR orchestrates diverse physiological processes. Intracellular Ca^2+^ signaling constitutes a central regulatory network controlling processes such as proliferation [23], cell death [24], migration [25], immune response [26], and senescence [27, 28]. In cancer, dysregulated Ca^2+^ signaling creates both oncogenic dependencies and therapeutic vulnerabilities. Accumulating evidence indicates that intracellular Ca^2+^ levels increase in response to various senescence‐inducing stimuli [29, 30, 31], while calcium chelation using BAPTA‐AM can suppress multiple senescence‐associated phenotypes [32]. Notably, ER‐derived Ca^2+^ flux into the cytosol promotes mitochondrial calcium uptake, which enhances electron transport chain activity and drives mtROS production [33, 34]. This Ca^2+^‐dependent ROS accumulation links calcium signaling to oxidative stress, DNA damage responses, and the enforcement of cellular senescence [35]. Despite these insights, the upstream signals that engage cADPR‐mediated calcium release to initiate mtROS‐dependent senescence, particularly in cancer cells, remain poorly defined.

In this study, we identify triosephosphate isomerase 1 (TPI1) as a critical metabolic regulator that restrains cellular senescence through control of a previously unrecognized metabolite‐driven redox signaling axis. Using a CRISPR‐Cas9 metabolic screen in clear cell renal cell carcinoma (ccRCC), we demonstrate that TPI1 depletion leads to DHAP accumulation, which activates a SARM1–cADPR–Ca^2+^ pathway to drive mitochondrial ROS production. This Ca^2+^‐dependent redox stress promotes DNA damage and enforces p53–p21‐mediated senescence. Our findings uncover a mechanistic link between glycolytic metabolite accumulation and mitochondrial redox control and highlight a metabolite‐driven vulnerability that may be therapeutically exploitable in cancer.

## 2 Materials and Methods

### 2.1 Reagents

DHA (D189238), GAP (D347018), and MitoPY1 (M286913) were purchased from Aladdin. MitoSO Red (S0061S) was purchased from Beyotime. Mito‐TEMPO (HY‐112879), NAC (HY‐B0215), BAPTA‐AM (HY‐100545), EGTA (HY‐D0861), cADPR (HY‐N7395), 8‐Br‐ADPR (HY‐134261) and Rapamycin (HY‐10219) were purchased from MedChemExpress.

### 2.2 Cell Culture

Human renal cell carcinoma lines A498 (HTB‐44, ATCC), OS‐RC‐2 (CL‐0177, Procell), 769‐P (CRL‐1933, ATCC), 786‐O (CRL‐1932, ATCC), Caki‐1 (HTB‐46, ATCC) and ACHN (CRL‐1611, ATCC), prostate cancer C4‐2 (CRL‐3314, ATCC), lung cancer A549 (CRM‐CCL‐185, ATCC), and bladder cancer T24 (HTB‐4, ATCC) cells were authenticated by short tandem repeat profiling. Cells were maintained in MEM (A498 and ACHN), RPMI‐1640 (OS‐RC‐2, 769‐P, 786‐O and C4‐2), McCoy's 5A (Caki‐1), F‐12K (A549) or DMEM (T24) supplemented with 10% fetal bovine serum (Procell) and 1% penicillin–streptomycin (Procell) at 37°C in 5% CO_2_. All cell lines were routinely confirmed negative for *Mycoplasma* contamination.

### 2.3 Metabolic CRISPR‐Cas9 Library Screening

A customized metabolic gene library consisting of a total of 6,896 sgRNAs—6,596 sgRNAs targeting 1,649 metabolic enzymes and regulators (4 sgRNAs per gene) and 300 control sgRNAs (comprising 80 sgRNAs targeting safe sites and 220 nontargeting sgRNAs)—was synthesized (Twist Bioscience) and cloned into lentiCRISPRv2 (Addgene #52961). Lentiviral particles were produced in HEK293T cells using psPAX2 and pMD2.G packaging plasmids. For the screen, A498 cells were independently infected at an MOI of 0.3 to achieve ∼30% transduction efficiency, maintaining an average sgRNA representation of >500 cells per sgRNA. Infected cells were selected with puromycin (2 µg/mL) for 21 days. All screens were performed in three independent biological replicates. Genomic DNA was extracted from cells collected at day 0 (reference baseline) and day 21 post‐infection using the DNeasy Blood & Tissue Kit (Qiagen, Cat. No. 69506). The sgRNA cassettes were amplified by PCR using the One‐step CRISPR NGS Library Construction Kit (Yomebio, Cat. No. PK201) and subjected to next‐generation sequencing on an Illumina NovaSeq platform, with a targeted sequencing depth of >500‐fold average coverage per sgRNA. Sequencing data were analyzed using MAGeCK v0.5.9. Raw reads were processed with the mageck count command to generate sgRNA count tables. Normalization and differential abundance analysis were performed using the mageck test command, which employs a negative binomial model to compare sgRNA counts between day 21 and day 0 samples across biological replicates. Gene‐level significance was determined by the robust rank aggregation (RRA) algorithm. Significantly enriched or depleted genes were defined based on the thresholds of |log_2_ fold change| ≥ 1 and FDR ≤ 0.05.

### 2.4 Plasmid Construction and Transduction

Short hairpin RNA sequences against human TPI1, TKFC, and SARM1 were synthesized by Miaolingbio.Inc and were cloned into the pLV3 vector. The human TPI1, TPI1(K14 mutated), and SARM1 cDNA were synthesized by miaolingbio.Inc, to construct the overexpression plasmid. The lentiviral vector was co‐transfected with PAX2 and VSVG packaging plasmids into HEK293T cells. The supernatant after 48 or 72 h of transfection was collected and concentrated overnight at 4°C according to the instructions of the lentivirus concentration reagent (GenStar). In order to construct a stable transfected cell line, the cells were infected with the corresponding lentivirus for 48 h, and the corresponding time was screened with the corresponding antibiotics.

### 2.5 Cell Viability Assays

For cell proliferation assays, tumor cells were seeded in 96‐well plates at 2000 cells per well in 100 µL complete medium. Cell viability was measured at the indicated time points using Cell Counting Kit‐8 (CK04, Dojindo) according to the manufacturer's instructions. For drug treatment experiments, assays were performed after treatment.

### 2.6 Edu Assays

For EdU incorporation assays, cells were seeded in 6‐well plates and cultured overnight. After indicated treatments, EdU (C0079S, Beyotime) was added to the medium at a final concentration of 10 µM and incubated for 2 h. Cells were fixed with 4% paraformaldehyde for 15 min at room temperature, permeabilized with 0.3% Triton X‐100 for 10–15 min and washed. The Click reaction was performed using Click reaction buffer, CuSO_4_, fluorescent azide, and Click Additive Solution according to the manufacturer's protocol (30 min, room temperature, protected from light). Cells were then counterstained with Hoechst 33342 (1:1000) for 10 min at room temperature in the dark, washed three times, and analyzed by fluorescence microscopy.

### 2.7 Colony Formation Assay

Cells were plated in 6‐well plates at a density of 1000 cells/well and cultured for 2 weeks. The cells were fixed with 4% paraformaldehyde and then stained with 0.5% crystal violet to count the colonies. All experiments were repeated three times, and the results were analyzed by ImageJ.

### 2.8 Quantitative Real‐Time PCR

Total RNA was extracted from cancer cells using the FastPure Cell/Tissue Total RNA Extraction Kit V2 (RC112‐01, Vazyme) following the manufacturer's protocol. cDNA was synthesized using HiScript III RT SuperMix (R323‐01, Vazyme). qRT‐PCR was performed with ChamQ Universal SYBR qPCR Master Mix (Q712‐02, Vazyme) on a CFX96 Real‐Time PCR System (Bio‐Rad). Gene expression levels were normalized to ACTB, and fold changes were calculated. Primers are listed in Table S1.

### 2.9 Western Blotting Analysis

Cells were lysed in RIPA buffer (R0010, Solarbio) containing a protease inhibitor cocktail and PMSF (G2008, Servicebio) on ice for 1 h. Protein concentrations were determined using a BCA Protein Assay Kit (P0009, Beyotime). Samples were boiled in 5× loading buffer (G2075, Servicebio) at 95°C for 10 min, separated by SDS‐PAGE, and transferred to PVDF membranes. After blocking with 5% skim milk, membranes were incubated with primary antibodies against Beta Actin (Proteintech, 66009‐1‐Ig), TPI1 (Proteintech, 10713‐1‐AP), TKFC (Proteintech, 12224‐1‐AP), LMNB1 (Proteintech, 66095‐1‐Ig), P21 (selleck, F0170), SARM1 (Proteintech, 28625‐1‐AP), CD38 (Zenbio, R381299), S6K1 (Proteintech, 14485‐1‐AP), Phospho‐S6K1 (Proteintech, 28735‐1‐AP), p53 (Proteintech, 60283‐2‐Ig), Phospho‐Histone H2AX (ABclonal, AP0687) at 4°C overnight. Following three washes with TBST, membranes were incubated with HRP‐conjugated anti‐rabbit (Proteintech, SA00001‐2) or anti‐mouse (Proteintech, SA00001‐1) secondary antibodies at room temperature for 1 h. Protein bands were visualized using an ECL substrate kit with a QuickChemi 5200 Imaging System.

### 2.10 Transcriptomics and Metabolomics

RNA‐seq and metabolomics were performed by Majorbio Bio‐Pharm Technology (Shanghai, China). Total RNA was extracted using TRIzol reagent and sequenced on the NovaSeq ReagentKit. Differential expression analysis was performed using DESeq2 (|log2FC| > 1, FDR < 0.05). For metabolomics, metabolites were extracted and analyzed by LC‐MS/MS (UHPLC‐Q Exactive HF‐X, Thermo Fisher). Data were processed on the Majorbio Cloud Platform for peak annotation against KEGG and HMDB databases. Differentially expressed genes and metabolite abundance data are provided in Table S2.

### 2.11 Measurement of Intracellular DHAP Levels

Intracellular dihydroxyacetone phosphate (DHAP) levels in tumor cells were quantified using a fluorometric DHAP assay kit (ab197003, Abcam) according to the manufacturer's protocol. Briefly, tumor cell lysates were prepared in assay buffer and deproteinized prior to analysis. Samples were incubated with the reaction mixture at 37°C for 60 min, and fluorescence intensity was measured using a microplate reader (Ex/Em = 535/587 nm). DHAP levels were quantified based on a standard curve, normalized to total protein concentration determined by the BCA assay, and expressed relative to the control group.

### 2.12 Calcium Measurements

Cellular calcium content was measured using a Calcium Colorimetric Assay Kit (S1063S, Beyotime). Cells were lysed in lysis buffer (100–200 µL per well for 6‐well plates), centrifuged at 12 000 g for 5 min at 4°C, and supernatants were collected. Calcium standards (0–1.0 mM) were prepared, and the detection working solution was made fresh by mixing detection buffer and chromogenic solution (1:1). In a 96‐well plate, 50 µL of standards or samples were mixed with 150 µL of detection working solution and incubated for 5–10 min at room temperature protected from light. Absorbance at 575 nm was measured using a microplate reader, and calcium concentrations were calculated from a standard curve.

For intracellular calcium imaging, cells were washed three times with PBS and incubated with Fluo‐4 AM (0.5–5 µM) (S1061S, Beyotime) for 30 min at 37°C. After three additional washes with PBS, cells were incubated for 20–30 min to allow complete de‐esterification of the dye. Intracellular calcium levels were then visualized using a fluorescence microscope.

### 2.13 Senescence β‐Galactosidase Staining

According to the instructions of the Senescence β‐Galactosidase Staining Kit (C0602, Beyotime), cells were fixed for 15 min at room temperature, washed with PBS, and incubated with staining working solution overnight at 37°C (without CO_2_). Stained cells were visualized using a light microscope.

### 2.14 Flow Cytometry

Cells treated under specific conditions (shown in the figure legends) were harvested. For cell cycle analysis, 1 × 10^6^ cells were resuspended in 100–150 µL PBS, mixed with 1 mL DNA cell cycle detection reagent (CYT‐PIR‐25, Cytognos), incubated for 10 min in the dark, and analyzed by flow cytometry. For ROS detection, cells were incubated with 10 µM DCFH‐DA (diluted 1:1000 in PBS) (S0033S, Beyotime) for 20 min at 37°C, washed three times, and analyzed by confocal microscopy or flow cytometry. FlowJo software was used to analyze the results.

### 2.15 Immunofluorescence Staining

Cells were inoculated in a confocal dish and fixed after the indicated treatment. Cells were permeabilized with 0.3% TritonX‐100 for 5 min, blocked with 3% BSA for 30 min at RT, and incubated with MMP3 (Proteintech, 17873‐1‐AP), IL‐6 (Proteintech, 21865‐1‐AP), HMGB1 (Proteintech, 10829‐1‐AP), and Phospho‐Histone H2AX (ABclonal, AP0687) overnight at 4°C. On the second day, cells were incubated with fluorescent‐labelled secondary antibodies for 1 h. Nuclei were stained with DAPI for 15 min. Finally, fluorescent images of the cells were obtained using a confocal microscope.

### 2.16 Tumor Xenograft Assay

Four‐week‐old male athymic BALB/c nude mice were purchased from Gempharmatech. During the experiment, mice were randomly divided into the indicated groups (five mice per group). All mice were maintained at room temperature with free access to food and water with a 12‐h light/dark cycle in a barrier facility. For subcutaneous tumors, the mice were subcutaneously injected with 2 × 10^6^ tumor cells in 100 µL PBS. For BAPTA‐AM treatment, BAPTA‐AM (2.5 mg/kg) was administered peritumorally every 2–3 days after tumor establishment until the experimental endpoint. Control mice received an equal volume of DMSO vehicle.

### 2.17 Luminex Liquid Suspension Biochip Detection

Supernatants were collected from A498 and OS‐RC‐2 cells stably transfected with shVector or shTPI1 after 48 h. Inflammatory cytokines and chemokines (CCL11, FGF, G‐CSF, GM‐CSF, IFN‐γ, IL‐1β, IL‐1α, IL‐2, IL‐4, IL‐5, IL‐6, IL‐7, IL‐8, IL‐9, IL‐10, IL‐12, IL‐13, IL‐15, IL‐17, CXCL10, MCP‐1, MIP‐1α, MIP‐1β, PDGF, CCL5, TNF‐α, VEGF) were quantified using a Luminex protein biochip detection system (Wayen Biotech) according to the manufacturer's protocol, with data acquired on a Luminex 200 system.

### 2.18 ELISA

The cells were cultured under the specified conditions and collected. According to the manufacturer's instructions, human IL6 detection kit (03204H1, Jingmei Biotechnology), human IL8 detection kit (04713H1, Jingmei Biotechnology), human TNF detection kit (03277H1, Jingmei Biotechnology), and human cADPR detection kit (7278H1, Jingmei Biotechnology) were used to detect the contents of corresponding cytokines and metabolites.

### 2.19 Patient Cohorts

Tissue microarrays (TMAs) were constructed from 234 ccRCC tumors and 172 adjacent normal tissues (collected under IRB approval). TMA sections were deparaffinized, antigen‐retrieved (pH 6.0 citrate buffer), and stained with anti‐TPI1 (1:200) using the Dako EnVision+ System. Staining intensity was quantified as mean optical density using ImageJ software (National Institutes of Health). TCGA KIRC RNA‐seq and mutation data were accessed via cBioPortal. Statistical analysis used R v4.2.2.

### 2.20 Statistical Analysis

All in vitro experiments were performed with three independent biological replicates, defined as independently cultured cell batches prepared on different occasions rather than technical replicates or independent clonal isolates, unless otherwise stated. Data are presented as mean ± SD. Statistical significance was determined by a two‐tailed unpaired Student's t‐test for pairwise comparisons or one‐way ANOVA with Tukey's post‐hoc test for multiple groups. *p* < 0.05 was considered significant. Statistical analyses were performed using GraphPad Prism v9.5.

## 3 Results

### 3.1 TPI1 is a Critical Regulator of ccRCC Proliferation Identified by Metabolic CRISPR Screening

**FIGURE 1:**
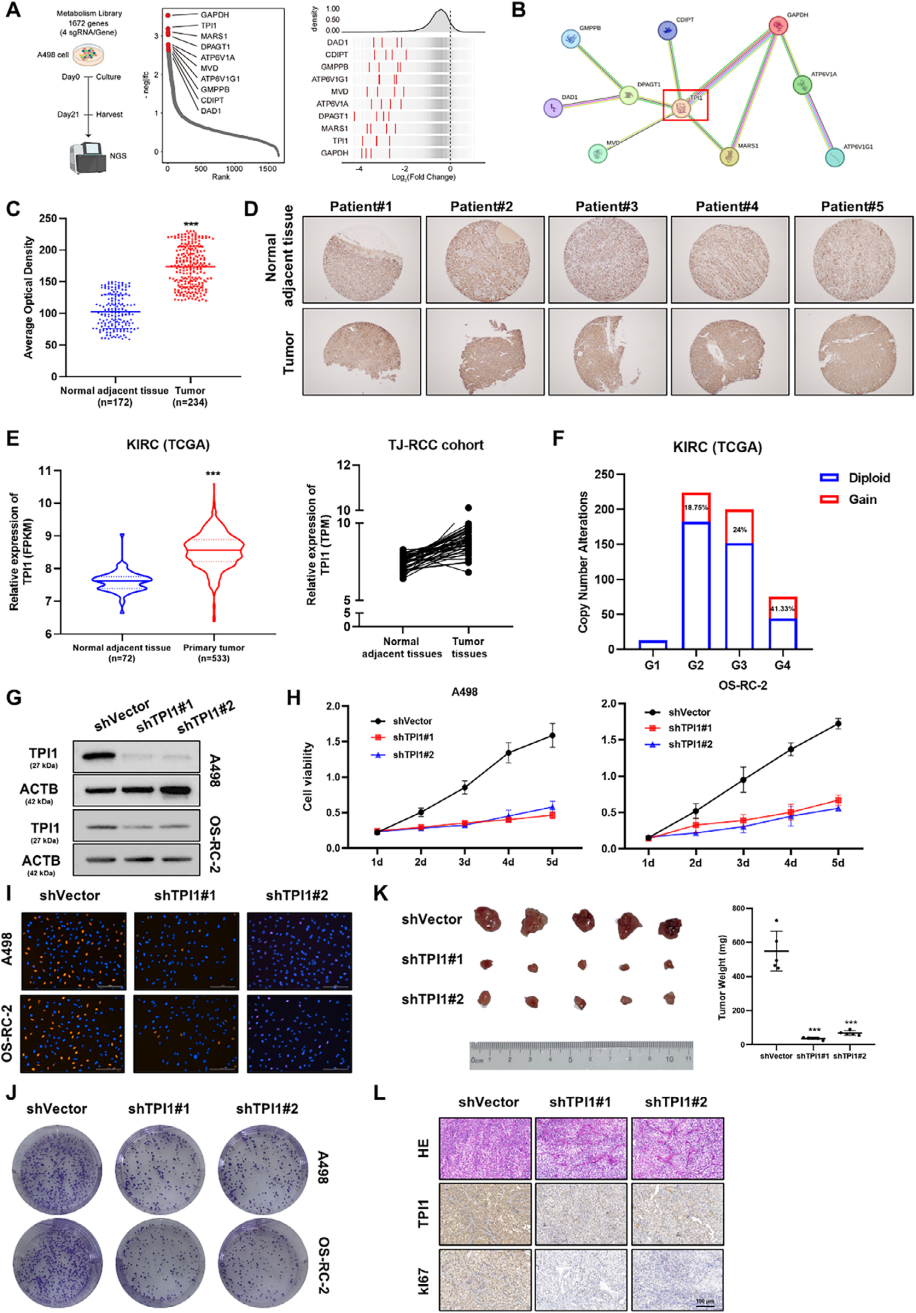
TPI1 is an oncogenic driver in ccRCC identified by CRISPR screening. (A) Schematic overview of A498 cell proliferation at day 21 following transduction with a CRISPR metabolic library (1,672 genes), with corresponding sgRNA abundance analysis. (B) Protein‐protein interaction network of the top 10 negatively selected genes. (C) Quantification of TPI1 immunohistochemical staining (average optical density) in tissue microarrays containing 172 adjacent normal and 234 tumor tissues from 301 patients. (D) Representative images of TPI1 immunohistochemical staining in paired samples. (E) TPI1 expression in tumor vs. adjacent normal tissues from TCGA (left) and the TJ‐RCC cohort (right). (F) Distribution of copy number alterations across tumor grades in TCGA KIRC. (G) Immunoblot validation of TPI1 knockdown in A498 and OS‐RC‐2 cells. (H–J) Cell proliferation was assessed by growth curves (H), EdU incorporation (I), and colony formation assays (J). (K) Representative images (left) and tumor weights (right) of xenografts derived from control or TPI1 knockdown OS‐RC‐2 cells. (L) Representative H&E, TPI1, and Ki67 staining of xenograft tumors (scale bar, 50 µm).

To identify essential metabolic targets in ccRCC, we performed a CRISPR screen in the cell line A498, using a metabolic gene library comprising 6688 sgRNAs (Figure 1A). TPI1 was robustly and significantly depleted on day 21, occupying a hub position among the top 10 depleted genes on day 21 (Figure 1B). Given its poorly characterized role in ccRCC, we prioritized it for detailed investigation. In the 301‐cohort comprising 234 tumor and 172 para‐cancerous tissue specimens, tissue microarray‐based immunohistochemistry demonstrated high TPI1 expression in renal cancer tissues (Figure 1C,D). External validation using TCGA and TJ‐RCC cohorts confirmed high TPI1 expression in tumor tissues (Figure 1E). TCGA data revealed that TPI1 expression increased with tumor grade, concomitant with a rise in gain‐of‐function mutation frequency (Figure 1F; Figure S1A). TPI1 expression levels were comparable across various ccRCC cell lines (Figure S1B). Immunofluorescence analysis further demonstrated that TPI1 was predominantly localized in the cytoplasm of A498 and OS‐RC‐2 cells (Figure S1C). Efficient knockdown of TPI1 in A498 and OS‐RC‐2 cells was confirmed by western blot analysis (Figure 1G). Functionally, TPI1 depletion significantly impaired cell proliferation, as demonstrated by growth curve analysis (Figure 1H) and EdU incorporation assay (Figure 1I). Moreover, clonogenic capacity was markedly reduced following TPI1 knockdown, as evidenced by colony formation assays (Figure 1J). In addition, TPI1 knockdown induced ∼10‐fold DHAP accumulation (Figure S1D). In vivo experiments recapitulated these findings, demonstrating that TPI1 knockdown significantly reduced tumor volume and weight and decreased Ki67 positivity, compared to controls (Figure 1K,L).

### 3.2 TPI1 Enzymatic Activity is Required for ccRCC Proliferation and Prevents DHAP Accumulation‐Associated Growth Suppression

**FIGURE 2:**
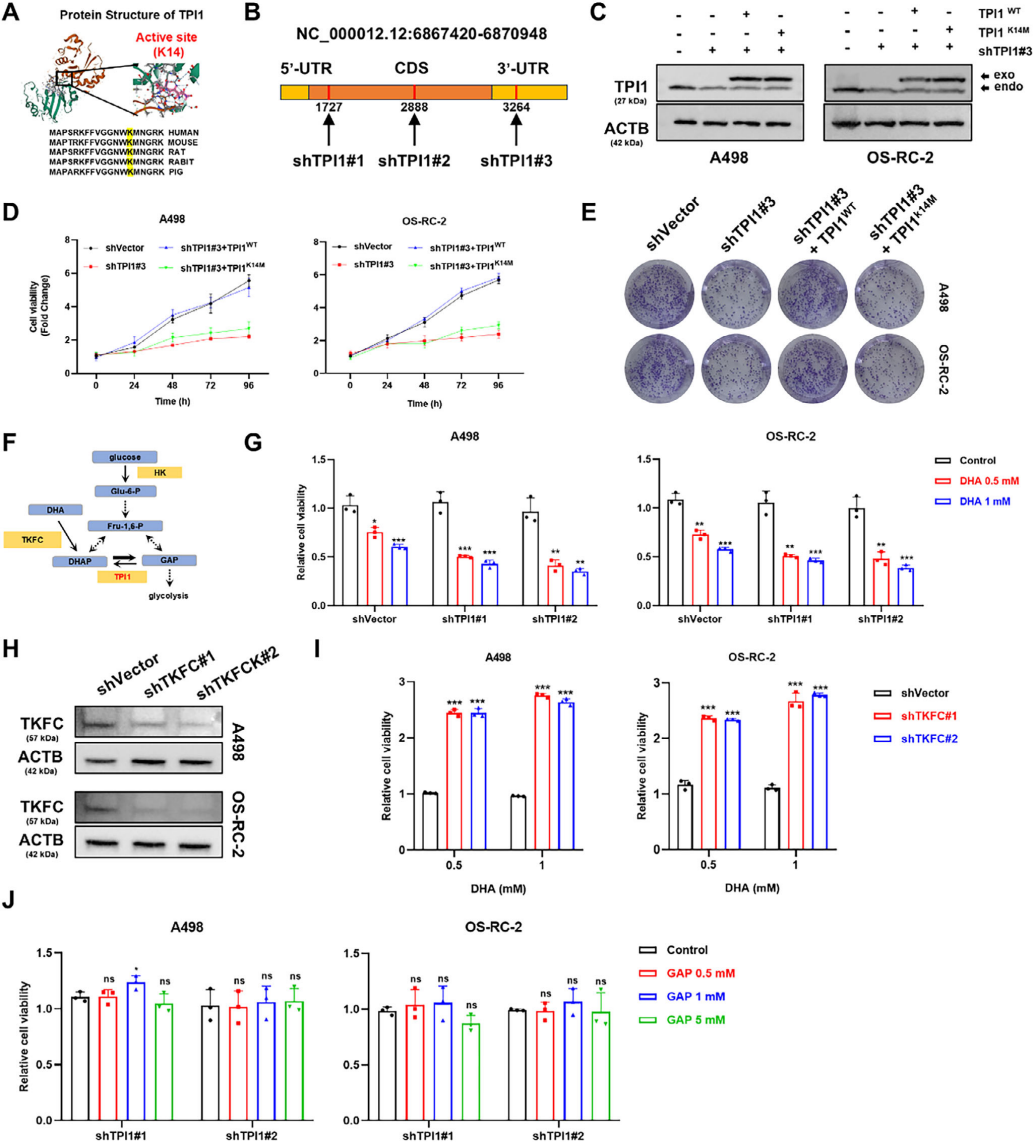
TPI1 enzymatic activity sustains ccRCC proliferation by limiting DHAP accumulation. (A) TPI1 protein structure (top) and cross‐species alignment of conserved catalytic residues (bottom). (B) Schematic of shTPI1#3 targeting the 3′UTR of endogenous TPI1. (C) Generation of A498 and OS‐RC‐2 cells expressing wild‐type (WT) or catalytically inactive K14M TPI1 following endogenous knockdown. (D) Cell proliferation was assessed by growth curves under the indicated conditions. (E) Cell proliferation was assessed by colony formation assays under the indicated conditions. (F) Schematic illustration of TPI1‐mediated conversion of DHAP to GAP. (G) Cell viability in control and TPI1 knockdown cells with or without dihydroxyacetone (DHA, 72 h) supplementation. (H) Immunoblot validation of TKFC knockdown. (I) Cell viability in TKFC knockdown cells treated with indicated concentrations of DHA (72 h). (J) Cell viability in TPI1 knockdown cells with or without indicated concentrations of GAP (72 h).

Lys14 is an evolutionarily conserved catalytic residue of TPI1 (Figure 2A). To deplete endogenous TPI1 without affecting exogenous wild‐type (WT) and Lys14‐mutant TPI1 (K14M) expression, we designed shRNA targeting the 3'UTR of endogenous TPI1 (shTPI1#3) and validated knockdown efficiency by immunoblotting (Figure 2B,C). WT, but not K14M, rescued the proliferative and clonogenic defects induced by TPI1 knockdown, demonstrating that TPI1 function requires its enzymatic activity (Figure 2D,E). TPI1 is a metabolic enzyme that catalyzes DHAP‐GAP isomerization, which predominantly proceeds unidirectionally from DHAP to GAP under physiological conditions (Figure 2F). DHAP does not readily cross cell membranes, and no known plasma membrane transporters for DHAP exist in eukaryotes [20]. To mimic DHAP accumulation, we supplemented cells with dihydroxyacetone (DHA), a membrane‐permeable DHAP precursor. Exogenous DHA supplementation reduced cell viability (Figure 2G), whereas knockdown of TKFC, the enzyme converting DHA to DHAP, attenuated this cytotoxic effect (Figure 2H,I). Conversely, exogenous supplementation with the downstream metabolite GAP in TPI1 knockdown cells showed no significant rescue effect (Figure 2J). Taken together, these results demonstrate that accumulation of upstream metabolites appears to be a major contributor to the effects elicited by TPI1 knockdown.

### 3.3 TPI1 Knockdown Induces mtROS‐dependent DNA Damage and Cellular Senescence in ccRCC

**FIGURE 3:**
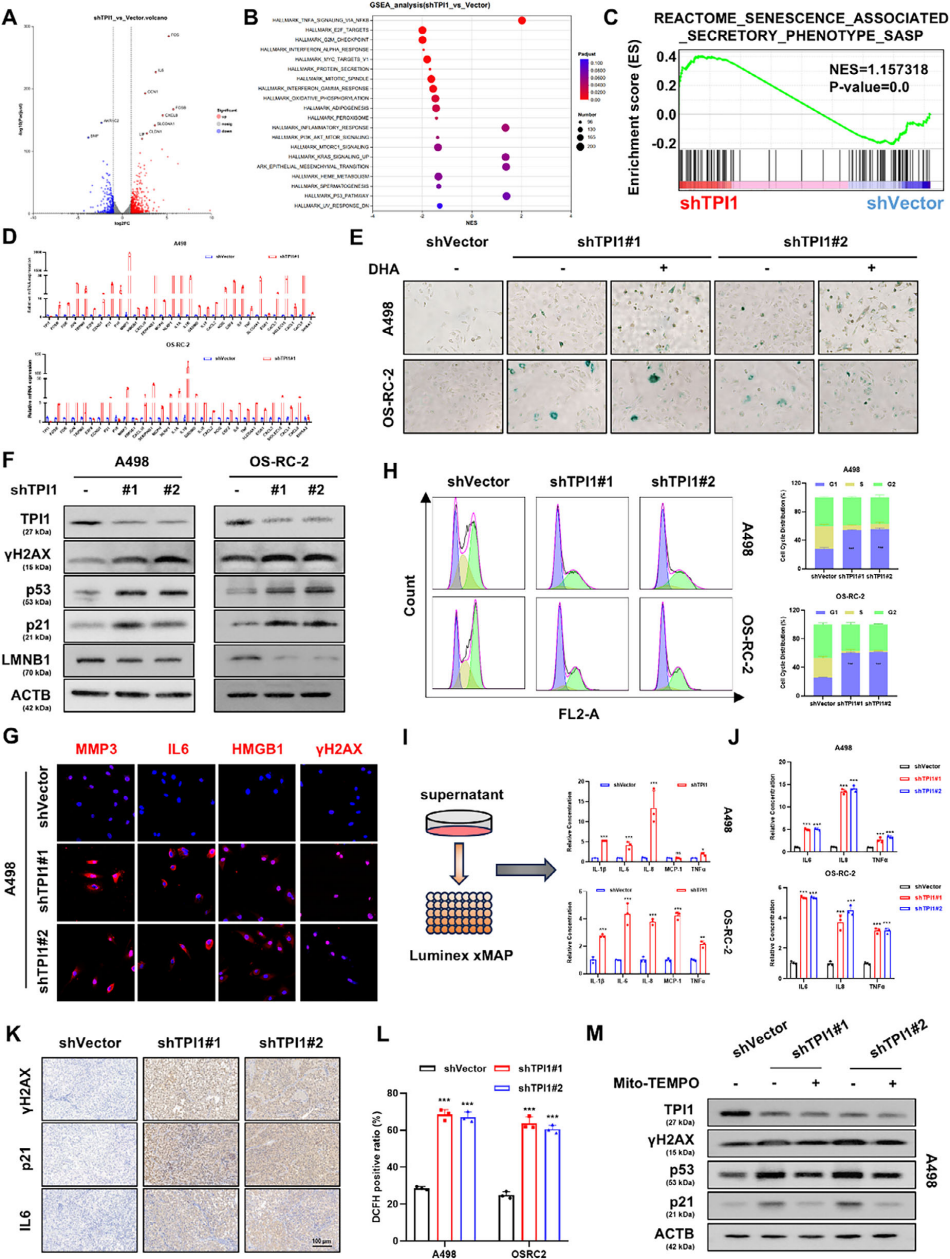
TPI1 knockdown induces cellular senescence. (A) Volcano plot of transcriptomic alterations in TPI1 knockdown A498 cells. (B, C) GSEA analysis showing enriched pathways (B) and SASP signatures (C). (D) qRT‐PCR validation of senescence‐associated genes. (E) SA‐β‐gal staining in control and TPI1 knockdown cells with or without DHA treatment (1 mM, 72 h). (F) Immunoblot analysis of γH2AX, p53, p21, and LMNB1 in control and TPI1 knockdown cells. (G) Immunofluorescence analysis of indicated proteins in control and TPI1 knockdown A498 cells. (H) Cell cycle distribution was analyzed by flow cytometry in control and TPI1 knockdown cells. (I) Cytokine secretion measured by Luminex in control and TPI1 knockdown cells. (J) Cytokine secretion measured by ELISA in control and TPI1 knockdown cells. (K) Immunohistochemical analysis of γH2AX, p21, and IL‐6 in xenografts. (L) ROS levels measured by flow cytometry in control and TPI1 knockdown cells. (M) Immunoblot analysis of indicated proteins in TPI1 knockdown A498 cells with or without Mito‐TEMPO (5 µM, 24 h) treatment.

To identify potential mechanistic changes, we performed transcriptome sequencing to examine global transcriptional alterations (Figure 3A). Gene set enrichment analysis (GSEA) revealed positive enrichment of TNF‐α signaling via NF‐κB, inflammatory response, and p53 pathway, while cell cycle‐related pathways including E2F targets and G2/M checkpoint were negatively enriched in TPI1 knockdown cells compared with controls (Figure 3B; Figure S2A). Given the enrichment patterns for cell cycle arrest and inflammatory pathways, we further characterized the transcriptome against the SASP gene set and observed positive enrichment in TPI1 knockdown cells (Figure 3C). Guided by transcriptomic findings, we validated selected results for cellular senescence, cell cycle, and inflammatory factors by PCR (Figure 3D). β‐galactosidase staining confirmed that TPI1 knockdown increased β‐galactosidase activity, a hallmark of cellular senescence, and this phenotype was further exacerbated by exogenous DHA supplementation (Figure 3E; Figure S2B). Immunoblotting revealed that TPI1 knockdown increased γH2AX, upregulated p53 and p21, and decreased LMNB1, indicating DNA damage, cell cycle arrest, and nuclear envelope damage, respectively (Figure 3F). Immunofluorescence analysis confirmed upregulation of senescence‐associated markers MMP3, IL‐6, HMGB1, and γH2AX in TPI1 knockdown cells (Figure 3G; Figure S2C). Flow cytometry analysis revealed significant G0/G1 cell cycle arrest in TPI1 knockdown cells (Figure 3H). Luminex multiplex analysis revealed significant upregulation of SASP components, including IL‐6, IL‐8, and GM‐CSF, upon TPI1 knockdown (Figure 3I; Figure S2D). ELISA further confirmed upregulation of IL‐6, IL‐8, and TNF‐α in TPI1 knockdown cells (Figure 3J). Immunohistochemistry revealed significant upregulation of γH2AX, p21, and IL‐6 in the TPI1 knockdown tumors (Figure 3K). Flow cytometry analysis revealed significant upregulation of ROS in TPI1 knockdown cells (Figure 3L; Figure S2E). Given that mtROS represents a major source of intracellular ROS, we next assessed mitochondrial superoxide and hydrogen peroxide levels using MitoSOX (Figure S2F) and MitoPY1 staining (Figure S2G), respectively. TPI1 knockdown markedly increased mitochondrial ROS accumulation in A498 and OS‐RC‐2 cells. Treatment with the mitochondrial‐targeted antioxidant Mito‐TEMPO significantly attenuated the elevated intracellular ROS induced by TPI1 depletion (Figure S2H). Moreover, Mito‐TEMPO treatment reduced the proportion of senescence‐associated β‐galactosidase‐positive cells following TPI1 knockdown (Figure S2I), accompanied by decreased γH2AX, p53, and p21 (Figure 3M; Figure S2J,K). Notably, the rescue effects of Mito‐TEMPO were comparable to those of NAC in reversing TPI1 knockdown‐induced γH2AX, p53, and p21 (Figure S2L). These findings suggest that mtROS acts as a critical downstream effector of TPI1 deficiency, prompting us to investigate the upstream signaling events responsible for ROS induction.

### 3.4 cADPR‐Dependent Calcium Signaling Contributes to TPI1 Knockdown‐Induced Phenotypes

**FIGURE 4:**
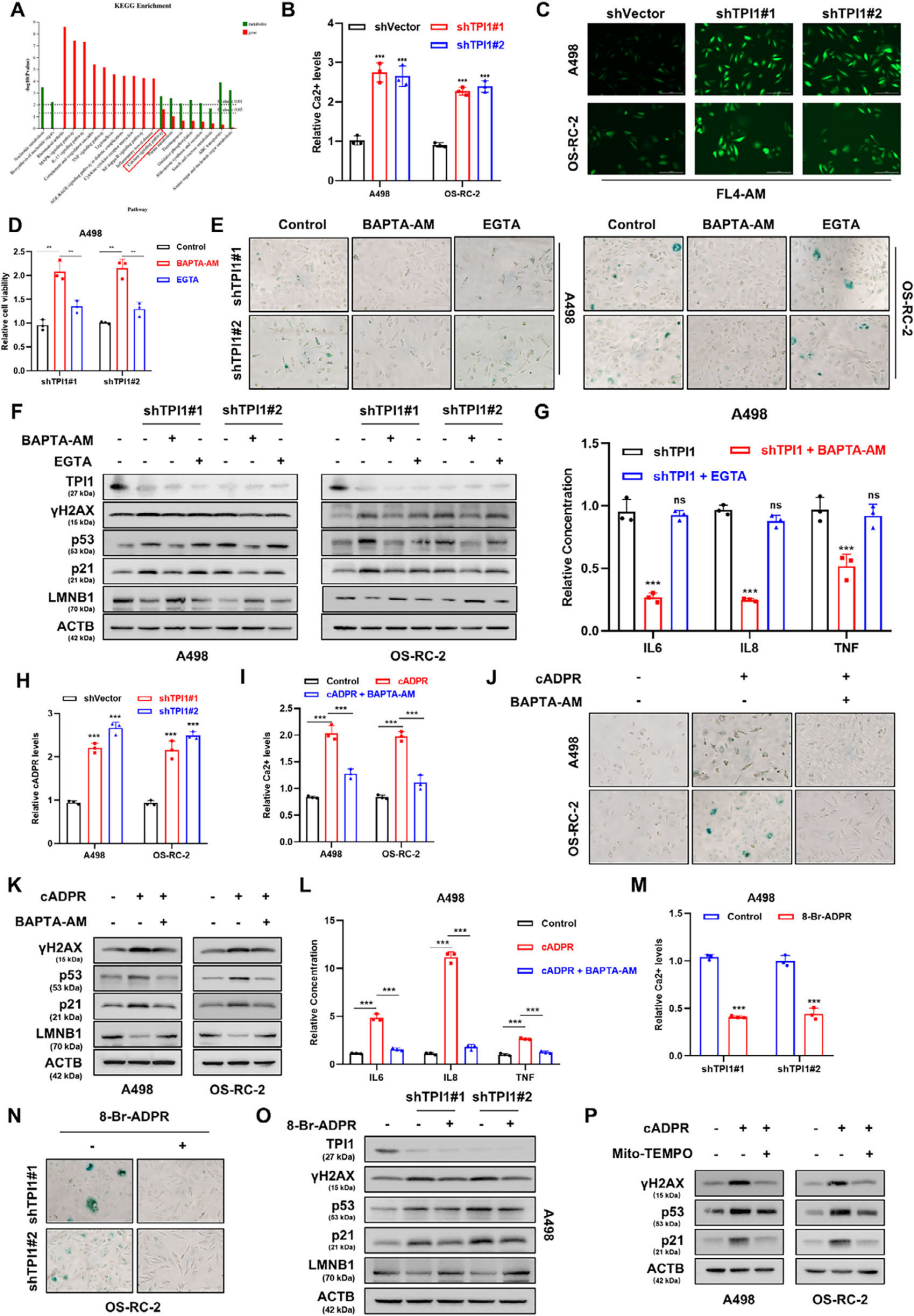
TPI1 knockdown induces senescence via cADPR‐mediated calcium release. (A) Integrated metabolomic and transcriptomic pathway analysis. (B) Intracellular calcium levels in control and TPI1 knockdown cells were measured by colorimetric assay. (C) Intracellular calcium levels in control and TPI1 knockdown cells were measured by Fluo‐4 AM staining. (D) Cell viability in TPI1‐knockdown A498 cells treated with BAPTA‐AM (2 µM, 48 h) or EGTA (0.5 mM, 48 h). (E) SA‐β‐gal staining in TPI1‐knockdown cells treated with BAPTA‐AM (2 µM, 48 h) or EGTA (0.5 mM, 48 h). (F) Immunoblot analysis of indicated proteins in TPI1‐knockdown cells treated with BAPTA‐AM (2 µM, 48 h) or EGTA (0.5 mM, 48 h). (G) Cytokine secretion in TPI1‐knockdown A498 cells treated with BAPTA‐AM (2 µM, 48 h) or EGTA (0.5 mM, 48 h). (H) Intracellular cADPR levels in control and TPI1 knockdown cells. (I) Intracellular calcium levels in cells treated with cADPR (100 nM, 48 h) and/or BAPTA‐AM (2 µM, 48 h). (J) SA‐β‐gal staining in cells treated with cADPR (100 nM, 48 h) and/or BAPTA‐AM (2 µM, 48 h). (K) Immunoblot analysis of indicated proteins in cells treated with cADPR (100 nM, 48 h) and/or BAPTA‐AM (2 µM, 48 h). (L) Cytokine secretion in A498 cells treated with cADPR (100 nM, 48 h) and/or BAPTA‐AM (2 µM, 48 h). (M) Intracellular calcium levels in TPI1‐knockdown A498 cells treated with or without 8‐Br‐cADPR (100 µM, 48 h). (N) SA‐β‐gal staining in TPI1‐knockdown OS‐RC‐2 cells treated with or without 8‐Br‐cADPR (100 µM, 48 h). (O) Immunoblot analysis of indicated proteins in TPI1‐knockdown A498 cells treated with or without 8‐Br‐cADPR (100 µM, 48 h). (P) Immunoblot analysis of indicated proteins in cells treated with cADPR (100 nM, 48 h) with or without Mito‐TEMPO (5 µM, 48 h).

Integrated metabolomic and transcriptomic analysis identified calcium signaling as a robustly enriched pathway (Figure 4A). We confirmed that TPI1 knockdown elevated intracellular calcium levels using calcium assays (Figure 4B) and fluorescent calcium probes (Figure 4C). To determine whether TPI1 knockdown‐induced loss of cell viability was mediated by intracellular or extracellular calcium, we treated cells with BAPTA‐AM, an intracellular chelator, or EGTA, an extracellular chelator (Figure S3A) [36]. BAPTA‐AM, but not EGTA, significantly rescued the impaired cell viability caused by TPI1 knockdown (Figure 4D; Figure S3B). Moreover, compared with EGTA, BAPTA‐AM markedly attenuated senescence‐associated phenotypes, including β‐galactosidase staining (Figure 4E; Figure S3C), DNA damage, p53/p21 activation, nuclear envelope disruption (Figure 4F), and the secretion of SASP factors (IL‐6, IL‐8, and TNF) (Figure 4G; Figure S3D). Consistently, in vivo peritumoral administration of BAPTA‐AM significantly increased the growth of TPI1‐knockdown tumors compared with DMSO (Figure S3E), further supporting a critical role for intracellular Ca^2+^ accumulation in mediating TPI1 loss‐induced tumor suppression. Metabolomic analysis revealed that cADPR, a mediator of ER calcium release, was elevated in the TPI1 knockdown group, prompting us to further investigate the role of the cADPR‐calcium signaling axis in cellular senescence (Figure S3F). Data from Ralph's team demonstrated that cADPR expression was lower in renal cancer tissues compared to normal kidney (Figure S3F) [37]. Both TPI1 knockdown and DHA supplementation induced cADPR upregulation (Figure 4H; Figure S3G). Treatment with cADPR increased intracellular calcium levels, an effect that was reversed by BAPTA‐AM (Figure 4I). Furthermore, treatment with cADPR alone was sufficient to induce senescence‐associated phenotypes, as evidenced by increased β‐galactosidase activity (Figure 4J; Figure S3H), enhanced DNA damage, activation of p53/p21 signaling, and nuclear envelope disruption (Figure 4K), accompanied by elevated secretion of SASP factors (IL‐6, IL‐8, and TNF) (Figure 4L; Figure S3I). Notably, co‐treatment with BAPTA‐AM markedly attenuated these cADPR‐induced senescence phenotypes, supporting a critical role for intracellular Ca^2+^ signaling in mediating the downstream effects of cADPR. Moreover, pharmacological inhibition of cADPR signaling using 8‐Br‐cADPR, a cell‐permeable cADPR antagonist, markedly attenuated the intracellular Ca^2+^ elevation induced by TPI1 knockdown (Figure 4M; Figure S3J) [38]. Correspondingly, 8‐Br‐cADPR significantly reduced senescence‐associated β‐galactosidase positivity (Figure 4N; Figure S3K,L), DNA damage, p53/p21 pathway activation, and nuclear envelope disruption (Figure 4O; Figure S3M), accompanied by decreased secretion of SASP factors (IL‐6, IL‐8, and TNF) (Figure S3N). These findings further support cADPR‐mediated Ca^2+^ signaling as a critical downstream effector of TPI1 loss‐induced senescence. Mito‐TEMPO treatment attenuated cADPR‐induced ROS production and DNA damage, and downregulated p53 and p21 expression, indicating that cADPR–Ca^2+^‐driven senescence is mediated, at least in part, by mtROS. (Figure 4P; Figure S3O). In summary, TPI1 deficiency was associated with elevated cADPR levels, which were accompanied by intracellular calcium elevation and subsequently promoted ROS‐mediated cellular senescence, a process that could be blocked by both calcium chelators and cADPR antagonists.

### 3.5 SARM1 Mediates TPI1 Deficiency‐Induced Senescence Through cADPR–Ca^2+^ Signaling and Oxidative Stress

**FIGURE 5:**
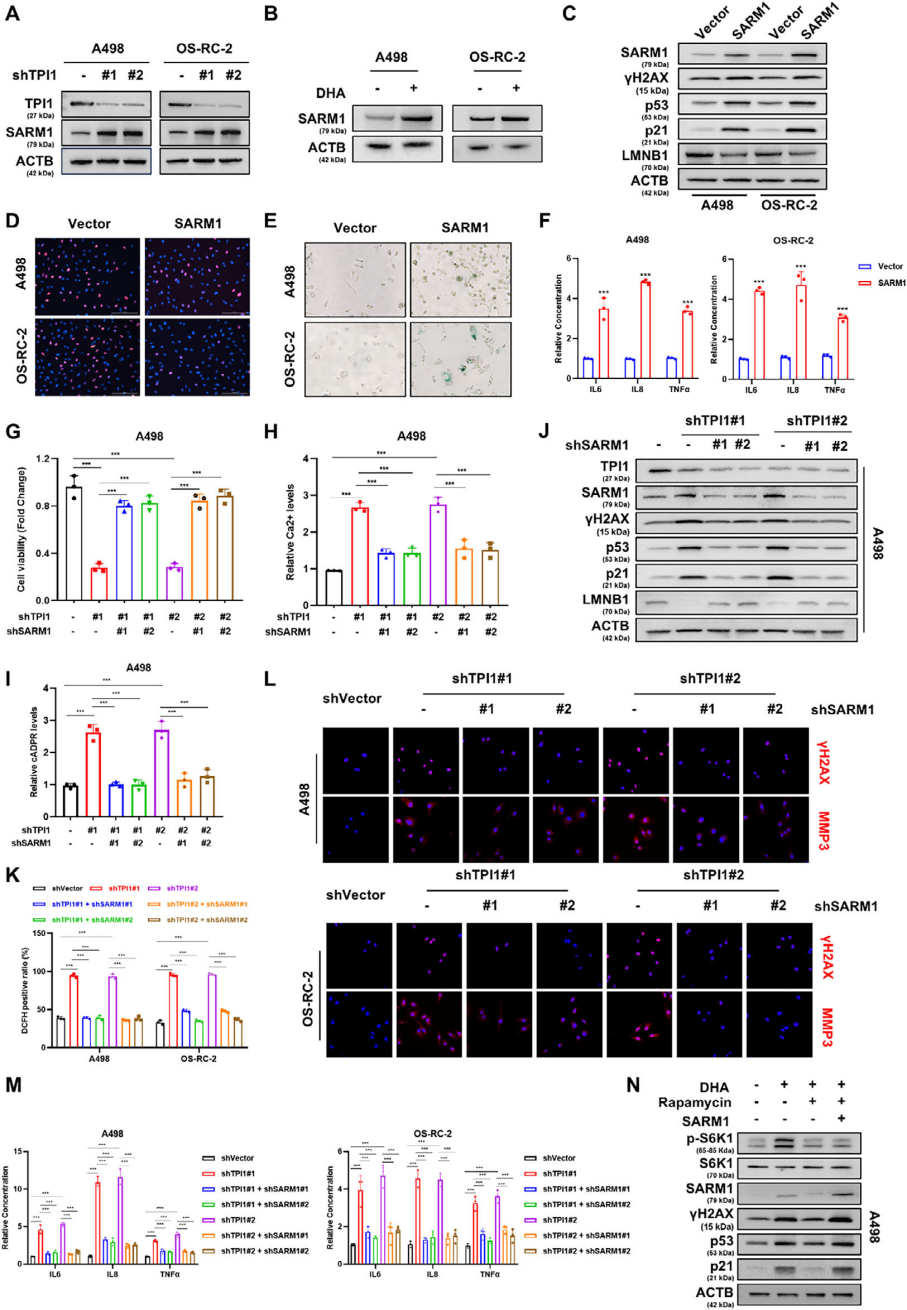
SARM1 is required for TPI1 knockdown‐induced senescence. (A) SARM1 expression in control and TPI1 knockdown cells after DHA treatment (1 mM, 72 h). (B) SARM1 expression in cells treated with DHA (1 mM, 72 h). (C) Immunoblot analysis of indicated proteins in control and SARM1‐overexpressing cells. (D) EdU incorporation assay in control and SARM1‐overexpressing cells. (E) SA‐β‐gal staining in control and SARM1‐overexpressing cells. (F) Cytokine secretion in control and SARM1‐overexpressing cells. (G) Cell viability in control, TPI1 knockdown, and TPI1/SARM1 double‐knockdown A498 cells. (H) Intracellular calcium levels in control, TPI1 knockdown, and TPI1/SARM1 double‐knockdown A498 cells. (I) Intracellular cADPR levels in control, TPI1 knockdown, and TPI1/SARM1 double‐knockdown A498 cells. (J) Immunoblot analysis of indicated proteins in control, TPI1 knockdown, and TPI1/SARM1 double‐knockdown A498 cells. (K) ROS levels measured by flow cytometry in control, TPI1 knockdown, and TPI1/SARM1 double‐knockdown cells. (L) Immunofluorescence analysis of indicated proteins in control, TPI1 knockdown, and TPI1/SARM1 double‐knockdown cells. (M) Cytokine secretion in control, TPI1 knockdown, and TPI1/SARM1 double‐knockdown cells. (N) Immunoblot analysis of the indicated proteins in A498 cells treated with DHA (1 mM, 72 h) with or without rapamycin (100 nM, 48 h) and SARM1 overexpression.

CD38 has long been considered the primary NAD^+^ consumer in mammals; however, recent studies have shown that SARM1 exhibits catalytic activity comparable to that of CD38 [39]. We therefore examined the protein levels of CD38 and SARM1 following TPI1 knockdown and found that SARM1 was markedly upregulated, whereas CD38 was undetectable (Figure 5A; Figure S4A). Exogenous DHA treatment similarly upregulated SARM1 expression (Figure 5B). We established SARM1 overexpression models to evaluate its impact on cellular senescence, which recapitulated the phenotype with elevated γH2AX, p53, and p21, and concomitant downregulation of LMNB1 (Figure 5C). SARM1 overexpression reduced cell viability (Figure S4B), clonogenic capacity (Figure S4C), and proliferation rate (Figure 5D). SARM1 overexpression increased β‐galactosidase activity (Figure 5E; Figure S4D), ROS production (Figure S4E) and SASP secretion (Figure 5F; Figure S4F). Further depletion of SARM1 in TPI1 knockdown cells partially rescued cell viability (Figure 5G; Figure S4G). Knockdown of SARM1 reversed the elevation of intracellular calcium (Figure 5H; Figure S4H) and cADPR induced by TPI1 knockdown (Figure 5I; Figure S4I). SARM1 knockdown reversed TPI1 knockdown‐induced upregulation of γH2AX, p53, and p21, as well as the downregulation of LMNB1 (Figure 5J; Figure S4J). Additionally, SARM1 knockdown suppressed TPI1 knockdown‐induced ROS production (Figure 5K; Figure S4K) and SASP secretion, including MMP3, IL‐6, IL‐8, and TNF (Figure 5L,M). Consistent with the in vitro findings, in vivo co‐depletion of SARM1 and TPI1 significantly increased tumor growth compared with TPI1 knockdown alone (Figure S4L), indicating that SARM1 deficiency partially rescues the tumor‐suppressive effect induced by TPI1 loss. Given that DHAP accumulation can activate mTORC1 signaling, we investigated whether mTORC1 contributes to SARM1 induction following TPI1 loss. Using S6K1 as a canonical downstream readout, immunoblotting revealed that TPI1 knockdown activated mTORC1 signaling, whereas treatment with rapamycin, a well‐established mTORC1 inhibitor, attenuated the upregulation of SARM1 induced by TPI1 depletion (Figure S4M). Consistently, in A498 and OS‐RC‐2 cells, DHA treatment increased S6K1, γH2AX, SARM1, and p53/p21 levels, indicative of mTORC1 activation, DNA damage, and senescence‐associated signaling. These DHA‐induced changes were markedly attenuated by rapamycin, suggesting that mTORC1 contributes to SARM1 upregulation and downstream responses. Notably, ectopic SARM1 expression in rapamycin‐treated cells still increased γH2AX, p53, and p21, indicating that SARM1 is sufficient to drive DNA damage and senescence‐associated signaling even under mTORC1 inhibition (Figure 5N; Figure S4N). Together, these findings support a role for mTORC1 in regulating SARM1 expression downstream of TPI1 deficiency. Collectively, these findings suggest that SARM1 functions as a critical mediator of TPI1 deficiency‐induced senescence, linking metabolic perturbations to calcium imbalance and oxidative stress, while mTORC1 may contribute to its regulation.

### 3.6 TPI1 Deficiency Induces Senescence Across Cancer Types in a SARM1‐ and cADPR–Ca^2+^‐Dependent Manner

**FIGURE 6:**
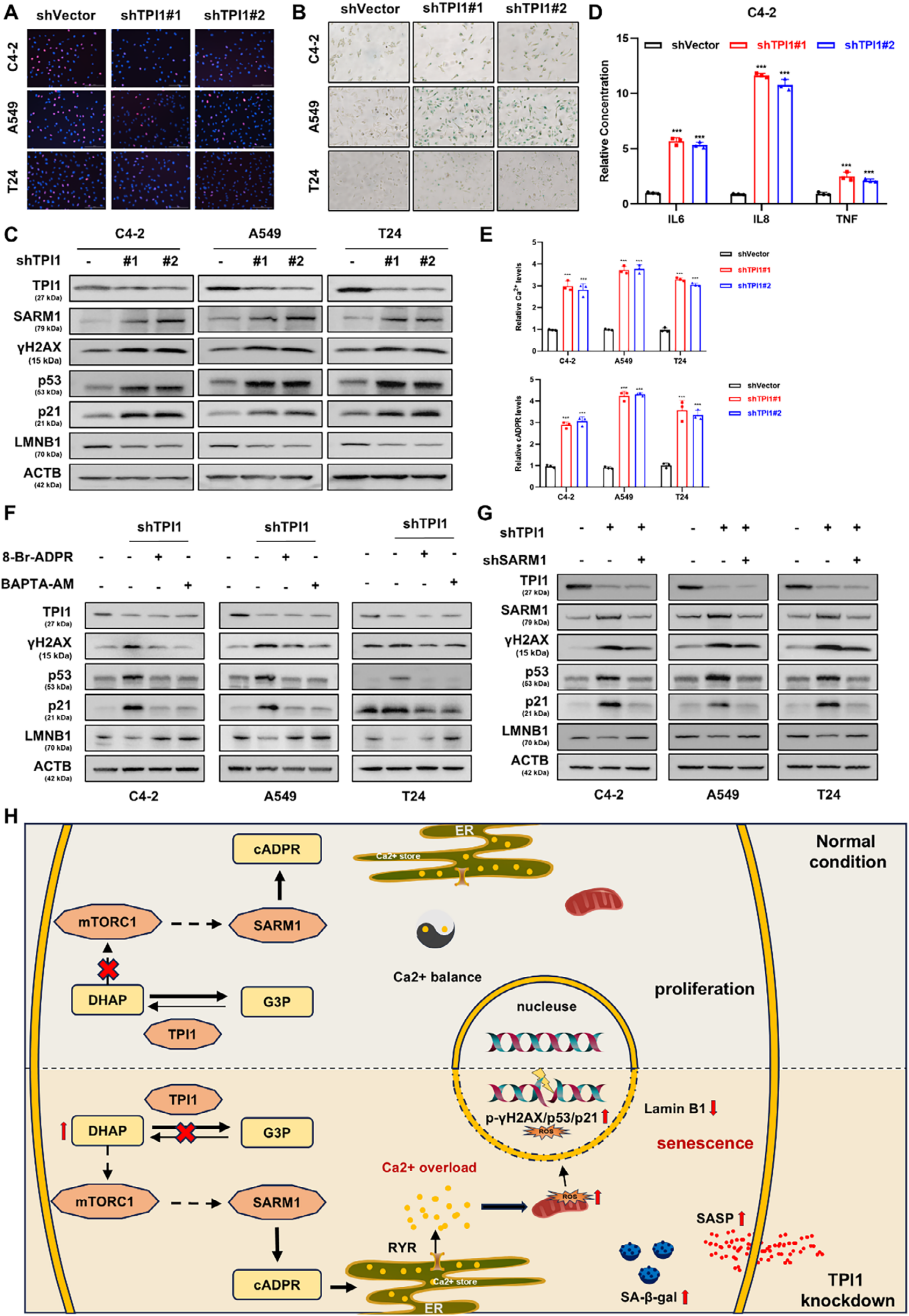
The TPI1‐SARM1‐cADPR pathway appears to be conserved across multiple cancer types. (A) EdU incorporation assay in control and TPI1 knockdown cells. (B) SA‐β‐gal staining in control and TPI1 knockdown cells. (C) Immunoblot analysis of indicated proteins in control and TPI1 knockdown cells. (D) Cytokine secretion in control and TPI1 knockdown C4‐2 cells. (E) Intracellular calcium (Top) and cADPR (Bottom) levels in control and TPI1 knockdown cells. (F) Immunoblot analysis of indicated proteins in cells treated with BAPTA‐AM (2 µM, 48 h) or 8‐Br‐cADPR (100 µM, 48 h). (G) Immunoblot analysis of indicated proteins in control, TPI1 knockdown, and TPI1/SARM1 double‐knockdown cells. (H) Proposed model illustrating TPI1 deficiency‐induced senescence via the SARM1‐cADPR‐calcium axis.

TPI1 is highly expressed across multiple tumor types (Figure S5A). Knockdown of TPI1 in prostate cancer C4‐2, lung cancer A549, and bladder cancer T24 cells reduced cell viability (Figure 6A) while elevating β‐galactosidase activity (Figure 6B; Figure S5B), upregulating SARM1, γH2AX, p53, and p21, downregulating LMNB1 (Figure 6C), and increasing IL‐6, IL‐8, and TNF secretion (Figure 6D; Figure S5C). In vivo, subcutaneous xenografts derived from TPI1‐knockdown T24 cells exhibited significantly reduced tumor growth compared with control tumors (Figure S5D). As expected, TPI1 knockdown increased intracellular cADPR and Ca^2+^ levels in C4‐2, A549, and T24 cells (Figure 6E). Consistent with a role for cADPR‐mediated Ca^2+^ signaling, chelation of intracellular Ca^2+^ with BAPTA‐AM or pharmacological antagonism of cADPR using 8‐Br‐cADPR attenuated the senescence‐associated phenotypes induced by TPI1 depletion, including β‐galactosidase activation (Figure S5E), γH2AX/p53/p21 upregulation, and LMNB1 downregulation (Figure 6F). Similarly, SARM1 knockdown markedly reversed TPI1 depletion‐induced β‐galactosidase activation (Figure S5F), as well as γH2AX/p53/p21 upregulation and LMNB1 reduction (Figure 6G).

The figure illustrates a mechanistic model in which TPI1 deficiency induces cellular senescence through activation of the cADPR‐calcium signaling axis. Under normal conditions, TPI1 catalyzes the interconversion between DHAP and G3P, thereby maintaining metabolic homeostasis, low SARM1 activity, and balanced intracellular calcium levels. Upon TPI1 knockdown, DHAP accumulation is associated with activation of mTORC1 signaling, which may contribute to SARM1 upregulation. Elevated SARM1 promotes cADPR production, leading to ryanodine receptor‐mediated calcium release from the endoplasmic reticulum and subsequent intracellular calcium overload. This calcium dysregulation enhances mitochondrial ROS generation, which triggers DNA damage, as evidenced by increased γH2AX and activation of the p53/p21 pathway. These events ultimately drive cellular senescence, characterized by nuclear envelope disruption (LMNB1 downregulation), elevated SA‐β‐gal activity, and increased secretion of SASP factors. (Figure 6H).

## 4 Discussion

In this study, we identify TPI1 as a critical metabolic regulator that constrains cellular senescence and uncover a previously unappreciated signaling axis linking glycolytic imbalance to calcium‐dependent redox stress. Through a combination of genetic and biochemical approaches, we demonstrate that loss of TPI1 does not primarily induce cell death, but instead drives a robust senescence phenotype across multiple cancer cell types, highlighting a noncanonical consequence of metabolic perturbation.

A central conceptual advance of this work is the repositioning of DHAP from a passive glycolytic intermediate to an active signaling metabolite [11, 40]. While DHAP has recently been implicated in the regulation of mTORC1 activity as an indicator of glucose availability [20, 41], our findings extend its functional scope by linking DHAP accumulation to the activation of a SARM1‐dependent signaling cascade. Importantly, the absence of a clearly defined molecular sensor for DHAP is not unique to our study but reflects a broader unresolved challenge in the field of metabolite signaling. Unlike amino acids or ATP, whose sensing mechanisms have been extensively characterized, DHAP lacks an established binding partner, and current models remain largely indirect. In this context, our data support a model in which DHAP accumulation functionally engages downstream signaling pathways, potentially through nutrient‐sensitive nodes such as mTORC1, thereby coupling metabolic state to stress‐responsive cellular outcomes.

Mechanistically, we show that activation of SARM1 leads to increased production of cyclic ADP‐ribose (cADPR), a well‐established second messenger that mobilizes intracellular Ca^2+^ stores via ryanodine receptors. Elevated cytosolic Ca^2+^ is known to profoundly impact mitochondrial function, and in this context, we find that Ca^2+^ overload promotes mitochondrial reactive oxygen species (mtROS) generation. Importantly, pharmacological scavenging of mtROS using Mito‐TEMPO markedly attenuates DNA damage and senescence markers, indicating that mtROS acts as a key downstream effector linking calcium dysregulation to genomic instability and cell fate determination. These findings support a coherent signaling cascade in which SARM1–cADPR–Ca^2+^–mtROS forms a functional axis driving senescence upon metabolic disruption.

In parallel, our data suggest that mTORC1 signaling contributes to this process. DHAP accumulation has been proposed to activate mTORC1 independently of canonical upstream inputs, and we observe that pharmacological inhibition of mTORC1 partially attenuates SARM1 expression and downstream senescence‐associated phenotypes. These results raise the possibility that mTORC1 functions as a modulatory node linking metabolic cues to stress signaling pathways. However, given the pleiotropic nature of mTORC1, further studies will be required to dissect whether its effects on SARM1 are direct or mediated through broader transcriptional or metabolic reprogramming.

From a broader perspective, our study highlights a multilayered connection between metabolism, calcium signaling, and redox homeostasis in the regulation of cellular senescence. While mitochondrial ROS has long been recognized as a driver of senescence, our findings place calcium signaling upstream of mtROS in the context of metabolic stress, providing a more integrated framework for understanding how intracellular signals converge on mitochondrial dysfunction. This is particularly relevant given the emerging appreciation that calcium fluxes and mitochondrial redox state are tightly coupled in determining cell fate under stress conditions.

Despite these advances, several important questions remain. First, the molecular mechanism by which DHAP is sensed and transduced into SARM1 activation is still unclear, and the identification of potential DHAP‐binding proteins or metabolic sensors will be critical for fully understanding this pathway. Although our data support a functional role for the SARM1–cADPR–Ca^2+^ axis in TPI1 loss‐induced senescence, whether this signaling cascade is fully reversible remains uncertain, particularly given the context‐dependent plasticity of senescence programs. Second, while our findings support a central role for mtROS in mediating senescence, we cannot exclude contributions from other ROS sources or parallel stress pathways, including broader metabolic or nutrient‐sensing networks that may act alongside SARM1 signaling. Moreover, because mTORC1 inhibition only partially attenuated downstream phenotypes, alternative interpretations remain possible, and SARM1 activation may represent either a direct consequence of DHAP accumulation or a secondary response to broader metabolic imbalance. Third, the in vivo relevance of this signaling axis, particularly in tumor progression and therapy response, warrants further investigation.

In summary, we propose a model in which TPI1 functions as a metabolic gatekeeper that restrains a DHAP‐driven signaling cascade linking SARM1 activation to calcium‐dependent mitochondrial oxidative stress and cellular senescence. This work not only expands the functional landscape of glycolytic metabolites but also provides mechanistic insight into how metabolic perturbations are translated into cell fate decisions. Targeting components of this axis may offer new opportunities for modulating senescence in cancer and other age‐related diseases.

## Author Contributions

**Chunyu Liu**: Conceptualization, methodology, investigation, writing – original draft, writing – review and editing. **Shun Wu**: Data curation, formal analysis, investigation, writing – review and editing. **Chuang Wang**: Data curation, formal analysis, investigation, writing – review and editing. **Zirui Zhou**: Conceptualization, resources, methodology, formal analysis, investigation, writing – review and editing. **Tianwei Cai**: Data curation, investigation, writing – review and editing. **Wen Tao**: Data curation, investigation, writing – review and editing. **Shidong Zuo**: Data curation, investigation, writing – review and editing. **Chi Zhang**: Data curation, investigation, writing – review and editing. **Yuhao Dong**: Data curation, investigation, writing – review and editing. **Yi Feng**: Data curation, investigation, writing – review and editing. **Qingbo Huang**: Data curation, investigation, writing – review and editing. **Baojun Wang**: Data curation, investigation, writing – review and editing. **Xin Ma**: Data curation, investigation, writing – review and editing. **Haoli Ma**: Conceptualization, resources, methodology, formal analysis, supervision, investigation, writing – review and editing. **Xu Zhang**: Conceptualization, resources, supervision, funding acquisition, writing – review and editing. **Yan Huang**: Conceptualization, resources, supervision, funding acquisition, project administration, writing – review and editing.

## Conflicts of Interest

The authors declare no conflicts of interest.

## Supporting information




**Supporting file 1**: advs76614‐sup‐0001‐SuppMat.docx.

**Supporting file 2**: advs76614‐sup‐0002‐Tables.xlsx.

## Data Availability

The data that supports the findings of this study are available in the supplementary material of this article.
