# Supplementary material for: TPI1 Loss Triggers a Metabolite‐Driven Mitochondrial Redox Vulnerability via the SARM1–cADPR–Ca2+ Axis
Source: Adv Sci (Weinh). 2026 Jul 17;13(56):e76614. doi: 10.1002/advs.76614 (PMC13379248; doi:10.1002/advs.76614)
Supplement: Supplementary file 1 [file ADVS-13-e76614-s001.docx]

**Supplementary Figures**

**Supplementary Figure 1. TPI1 is an oncogenic driver in ccRCC identified by CRISPR screening**

(**A**) TPI1 expression across tumor grades in TCGA KIRC.

(**B**) Immunoblot analysis of TPI1 expression in ccRCC cell lines.

(**C**) Subcellular localization of TPI1 in A498 and OS-RC-2 cells determined by immunofluorescence.

(**D**) Quantification of relative DHAP levels in A498 and OS-RC-2 cells following TPI1 knockdown using a DHAP assay kit.

**Supplementary Figure 2. TPI1 knockdown induces cellular senescence**

(**A**) GSEA of selected gene sets in TPI1 knockdown A498 cells.

(**B**) Quantification of SA-β-gal-positive cells in control and TPI1 knockdown cells with or without DHA treatment (1 mM, 72 h).

(**C**) Immunofluorescence analysis of indicated proteins in TPI1 knockdown OS-RC-2 cells.

(**D**) Cytokine secretion measured by Luminex xMAP analysis in control and TPI1 knockdown cells.

(**E**) ROS levels measured by flow cytometry in control and TPI1 knockdown cells.

(**F**) Flow cytometric analysis of mitochondrial superoxide levels in control and TPI1-knockdown cells using MitoSO™ Red kit.

(**G**) Fluorescence microscopy images of mitochondrial hydrogen peroxide levels in control and TPI1-knockdown cells detected by MitoPY1 staining.

(**H-J**) Effects of Mito-TEMPO (5 μM, 24 h) treatment on ROS levels (H), SA-β-gal staining (I) and DNA damage (J).

(**K**) Immunoblot analysis of indicated proteins in TPI1 knockdown OS-RC-2 cells with or without Mito-TEMPO (5 μM, 24 h) treatment.

(**L**) Immunoblot analysis of indicated proteins in TPI1 knockdown cells with or without Mito-TEMPO (5 μM, 24 h) or NAC (5 mM, 24 h) treatment.

**Supplementary Figure 3. TPI1 knockdown induces senescence via cADPR-mediated calcium release**

(**A**) Intracellular calcium levels in TPI1-knockdown cells treated with BAPTA-AM (2 μM, 48 h) or EGTA (0.5 mM, 48 h).

(**B**) Cell viability in TPI1-knockdown OS-RC-2 cells treated with BAPTA-AM (2 μM, 48 h) or EGTA (0.5 mM, 48 h).

(**C**) Quantification of SA-β-gal-positive cells in TPI1-knockdown cells treated with BAPTA-AM (2 μM, 48 h) or EGTA (0.5 mM, 48 h).

(**D**) Cytokine secretion in TPI1-knockdown OS-RC-2 cells treated with BAPTA-AM (2 μM, 48 h) or EGTA (0.5 mM, 48 h).

(**E**) Image of TPI1-knockdown OS-RC-2 xenografts following peritumoral administration of DMSO or BAPTA-AM.

(**F**) Metabolomic profiling of control and TPI1 knockdown cells (Left). And cADPR levels in normal kidney and renal tumor tissues (data provided by Ralph’s group) (Right).

(**G**) cADPR levels following DHA treatment (1 mM, 72 h) in TPI1-knockdown cells.

(**H**) Quantification of SA-β-gal-positive cells following cADPR (100 nM, 48 h) treatment with or without BAPTA-AM (2 μM, 48 h).

(**I**) Cytokine secretion in OS-RC-2 cells treated with cADPR (100 nM, 48h) and/or BAPTA-AM (2 μM, 48h).

(**J**) Intracellular calcium levels in TPI1 knockdown OS-RC-2 cells treated with 8-Br-cADPR (100 μM, 48 h).

(**K**) Quantification of SA-β-gal-positive cells following 8-Br-cADPR (100 μM, 48 h) in TPI1 knockdown OS-RC-2 cells.

(**L**) Effects of 8-Br-cADPR (100 μM, 48 h) on SA-β-gal staining in TPI1 knockdown A498 cells.

(**M**) Immunoblot analysis of DNA damage and senescence markers following 8-Br-cADPR (100 μM, 48 h) in TPI1 knockdown OS-RC-2 cells.

(**N**) Cytokine secretion in TPI1 knockdown cells treated with 8-Br-cADPR (100 μM, 48 h).

(**O**) Effects of cADPR (100 nM, 48 h) treatment with or without Mito-TEMPO (5 μM, 48 h) on ROS levels

**Supplementary Figure 4. SARM1 is required for TPI1 knockdown-induced senescence**

(**A**) Immunoblot analysis of CD38 expression in control and TPI1 knockdown A498 and OS-RC-2 cells.

(**B-C**) Effects of SARM1 overexpression on cell proliferation assessed by growth curves (B) and colony formation assays (C).

(**D**) Quantification of SA-β-gal-positive cells in control and SARM1-overexpressing cells.

(**E**) ROS levels in SARM1-overexpressing cells measured by flow cytometry.

(**F**) Immunofluorescence analysis of MMP3, IL-6, HMGB1, and γH2AX in SARM1-overexpressing cells.

(**G**) Cell viability in control, TPI1 knockdown, and TPI1/SARM1 double-knockdown OS-RC-2 cells.

(**H**) Intracellular calcium levels in control, TPI1 knockdown, and TPI1/SARM1 double-knockdown OS-RC-2 cells.

(**I**) Intracellular cADPR levels in control, TPI1 knockdown, and TPI1/SARM1 double-knockdown OS-RC-2 cells.

(**J**) Immunoblot analysis of indicated proteins in control, TPI1 knockdown, and TPI1/SARM1 double-knockdown OS-RC-2 cells.

(**K**) ROS levels in control, TPI1 knockdown, and TPI1/SARM1 double-knockdown cells.

(**L**) Image of subcutaneous xenograft tumors derived from TPI1-knockdown, or TPI1/SARM1 double-knockdown OS-RC-2 cells (Left). Quantification of xenograft tumor weights from the indicated groups (Right).

(**M**) Immunoblot analysis of indicated proteins in TPI1 knockdown cells treated with or without rapamycin (100 nM, 48 h).

(**N**) Immunoblot analysis of the indicated proteins in OS-RC-2 cells treated with DHA (1 mM, 72 h) with or without rapamycin (100 nM, 48 h) and SARM1 overexpression.

**Supplementary Figure 5. The TPI1-SARM1-cADPR pathway appears to be conserved across multiple cancer types**

(**A**) TPI1 expression in tumor and adjacent normal tissues (TCGA).

(**B**) Quantification of SA-β-gal-positive cells in control and TPI1 knockdown cells.

(**C**) Cytokine secretion in control and TPI1 knockdown A549 and T24 cells.

(**D**) Images of subcutaneous xenograft tumors derived from control or TPI1-knockdown T24 cells (Left). Quantification of tumor weights from the indicated groups (Right).

(**E**) Representative SA-β-gal staining images of TPI1-knockdown cells treated with BAPTA-AM (2 μM, 48 h) or 8-Br-cADPR (100 μM, 48 h) (Left). Quantification of SA-β-gal-positive cells from the indicated groups (Right).

(**F**) Representative SA-β-gal staining images of TPI1-knockdown or TPI1/SARM1 double-knockdown cells (Left). Quantification of SA-β-gal-positive cells from the indicated groups (Right).
